# HDL Particle Size and Functionality Comparison between Patients with and without Confirmed Acute Myocardial Infarction

**DOI:** 10.1155/2019/3074602

**Published:** 2019-03-03

**Authors:** Raissa de Miranda Teixeira, Nicole Cruz de Sá, Ana Paula Caires dos Santos, Vanessa Rocha Anjos e Silva, Elaine Christine de Magalhães Cabral Albuquerque, Luiz Claudio Lemos Correia, Ricardo David Couto

**Affiliations:** ^1^Clinical Chemistry and Lipid Metabolism Laboratory, Department of Clinical and Toxicological Analysis, Faculty of Pharmacy, Federal University of Bahia (UFBA), Salvador, Bahia, Brazil; ^2^Pharmacy Postgraduate Program (PPGFAR), Faculty of Pharmacy, Federal University of Bahia (UFBA), Salvador, Bahia, Brazil; ^3^Escola Bahiana de Medicina e Saúde Pública, Hospital São Rafael, Salvador, Bahia, Brazil; ^4^Naval Hospital of Salvador, Brazilian Marine Forces, Salvador, Bahia, Brazil; ^5^PEI, Industrial Engineering Program, Department of Chemical Engineering, Federal University of Bahia (UFBA), Salvador, Bahia, Brazil

## Abstract

**Introduction:**

Cardiovascular diseases (CVDs) continue to be the most common cause of death worldwide, and acute myocardial infarction (AMI) is noteworthy due to its great magnitude.

**Objectives:**

This study was carried out to evaluate the structure (molecular and particle size) and functionality of high-density lipoprotein (HDL) shortly after AMI, in the presence of acute inflammatory response.

**Casuistic and Methods:**

A cross-sectional, observational study was conducted between January 2015 and August 2016, with a total convenient sample of 85 patients. The patients' data were segregated according to the Registry of Acute Myocardial Infarction (REAMI), with 45 confirmed AMI patients. The study groups consisted of patients from both sexes, older than 35 years, presented to the Hospital São Rafael (HSR) initially with AMI clinical symptoms. In addition, 40 nonischemic control patients (CPs), without AMI symptomatology, and according to previous inclusion criteria, were selected for convenience in an outpatient care unit. The HDL particle size was measured by laser light scattering (LLS), after separation of HDL from apoB-rich lipoproteins. The paraoxonase-1 (PON-1) activity was determined in a spectrophotometer by using paraoxon as a substrate. The other laboratory marker information, secondary data, was obtained in the laboratory system.

**Results:**

The HDL particle size, free cholesterol, and hs-CRP analysis showed significant differences when compared between REAMI and CP groups (*p* < 0.0001, *p*=0.007, and *p* < 0.0001; two-tailed unpaired *t*-test, respectively). Regarding paraoxonase, the data comparison between REAMI and CP groups was also significantly different (*p* < 0.0067; two-tailed unpaired *t*-test).

**Conclusion:**

Despite an important current database on the HDL cholesterol role, our study provides relevant complementary information about the HDL particle susceptibility to the inflammation following AMI. The HDL particles' quantitative and functional attributes should be measured as markers of HDL functionality.

## 1. Introduction

Cardiovascular diseases (CVDs) continue to be the most common cause of death in the world [1], and acute myocardial infarction (AMI) is noteworthy due to its great magnitude. In 2011, about 20 million people suffered from cardiovascular diseases worldwide, of which approximately 12 million were fatal victims of AMI [2]. Acute myocardial infarction represents the main cause of death and disability, with coronary atherosclerosis being one of the main causes [3]. Atherosclerosis is a CVD characterized by chronic inflammation of the artery wall and consequent plaque formation [4], a process known to be associated with endothelium damage, cell activation, and the release of inflammatory and immune response mediators [5]. The atherosclerotic lesions are driven by a series of specific and dynamic cellular and molecular responses. In vulnerable patients, atherosclerosis develops through the influence of several conditions that alter the endothelium hemostasis, such as aging, smoking, systemic arterial hypertension, hypercholesterolemia, diabetes, and obesity [4].

In view of this, several studies show the inverse relationship between HDL antiatherosclerotic role and the incidence of cardiovascular events, especially that related to reverse cholesterol transport (RCT), has been increasingly characterized. In addition to this protective action, HDL is also associated with other important functions, such as antioxidant protection, mediation of cholesterol efflux, inhibition of cell adhesion molecule expression and leukocyte activation, and the induction of nitric oxide production [6]. The HDL particles are a heterogeneous group of lipoproteins with peculiar functional and metabolic characteristics [7]. The HDL heterogeneity is a consequence of the continuous particle structure remodeling by the interaction with several plasma factors. Remodeling can be defined as a set of processes that can change HDL size, surface charge, and particle composition [8, 9]. HDL and its subclasses are currently separated by various methods, all based on physicochemical property's differences (e.g., density, size, electrophoretic mobility, and apolipoprotein content) which are determined by lipid and/or protein concentrations in different particles [8]. Currently, the idea to measure HDL particle size has been widely diffused from the previous use of the laser light scattering (LLS) method to measure LDL particle size. With some modifications, Lima and Maranhão [10] proposed that LLS could be used to measure HDL particle size, after apoB-containing lipoprotein precipitation.

In addition to the important role in RCT, HDL also has antioxidant activity attributed to paraoxonase-1 (PON-1) activity, an enzyme that confers a greater antioxidant potential to lipoproteins, through the reduction of lipid peroxidation-accumulated products, especially those associated with LDL [11]. HDL-PON-1 activity has also been implicated in the improvement of macrophage's cholesterol efflux [12].

Therefore, as we can see, biomarkers that can be used to evaluate the HDL structure and functionality should be measured to help understand the lipoprotein alterations observed in patients shortly after acute myocardial infarction.

## 2. Objective

The aim of this study was to evaluate the high-density lipoprotein structure and functionality, by measuring biomarkers of the structure (i.e., free and esterified cholesterol), diameter by particle size, and functionality (i.e., PON-1 activity) in the occurrence of acute myocardial infarction.

## 3. Materials and Methods

A cross-sectional, observational study was performed between January 2015 and August 2016, with a voluntary involvement of 85 participants: 45 patients allocated from the Registry of Acute Coronary Syndrome-AMI (REAMI) (case group) and 40 nonischemic control patients (CPs) without any symptoms of an ischemic event presented to the Faculty of Pharmacy Clinical and Toxicological Analysis Laboratory. The mean age of case and control groups' participants was 65.6 and 58.3 years, respectively. The sample size was calculated by considering a chance of 40 patients with chest pain be selected in a critical ambulatory of 101 patients with a maximum expected loss of 1%. Following that assumption, the minimum required sample size to obtain significance would not be inferior to 78 subjects. The study sample size was calculated by assuming a sample (to estimate a sample size needed to find 40 cases) sufficient to obtain a statistical power of at least 80% (1 − *β*) capable of detecting differences of 18.16 units of paraoxonase-1 (PON-1) activity between patients with and without AMI, considering a 10% variation. The sample calculation was performed using WINPEPI software for Windows, version 11.48 (Joe Abramson, PEPI—Programs for EPIdemiologists).

To include patients' participation from the REAMI group, the research protocol number 037/2011 was approved by Hospital São Rafael Ethics Committee, Monte Tabor, on July 25, 2011, and followed the resolution 196/96 requirements from the National Health Council. To include participants from the CP group was approved another protocol with additive resolution (no. 029/2014) by the Federal University of Bahia Ethics Committee.

**Figure 1:**
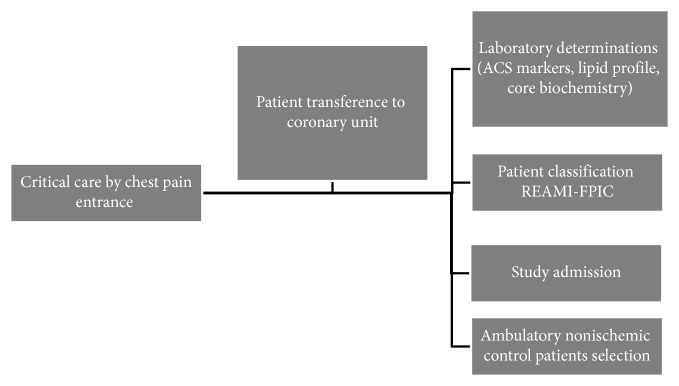
Patients' suspicious of ACS/AMI and CP inclusion into the study.

In order to include patients (cases and controls) in the study, the following inclusion criteria were used: to be included as REAMI, the patient should be with chest pain symptoms, or the patient should be with pain equivalent to that of ischemic event in the last 48 hours, with subsequent confirmation of obstructive coronary artery disease (CAD) greater than or equal to 50%. The CP group should not show any symptoms or complaints associated with an ischemic event and be a laboratory checkup participant (Figure 1). All patients were 35 years or older and freely accepted to participate in the study and signed a Free Prior and Informed Consent (FPIC).

## 4. Laboratory Determinations

Blood samples were collected in gel-separator tubes, centrifuged for 10 min at 3300 rpm, and then submitted to a laboratory routine. All patients had the lipid profile phenotypic classification for dyslipidemia (i.e., isolated hypercholesterolemia, only with the isolated increase of LDL-c, i.e., LDL-c ≥ 160 mg/dL; isolated hypertriglyceridemia, only with the isolated increase of TG: with fast-TG ≥ 150 mg/dL or without fast-TG ≥ 175 mg/dL; mixed hyperlipidemia, when LDL-c ≥ 160 mg/dL plus TG increasing, with fast-TG ≥ 150 mg/dL or without fast-TG ≥ 175 mg/dL; and low HDL-c, when isolated-HDL-c < 40 mg/dL (adult male) or <50 mg/dL (adult female) and could be also associated with the increasing of LDL-c and TG) [29]. The lipid profile determinations were performed by reflectance spectrophotometry (VITROS 5600 equipment, Johnson and Johnson), troponin and CK-MB mass by immunometric immunoassay, and PCR by reflectance spectrophotometry (VITROS 5600, Johnson and Johnson-USA). For CRP values below 6 mg/dL, samples were not diluted nor re-measured.

## 5. Paraoxonase Activity

The paraoxonase activity was performed by a spectrophotometric continuous kinetic reaction, according to the method described by Charlton-Menys et al. [13] and Gelisgen et al. [14]. The reading was made at 405 nm and 37°C using a 96-well SIRIO Microplate Reader Seac. SrL (Firenze, IT). The reaction is based on paraoxon hydrolysis with *p*-nitrophenol and diethyl phosphate formation. To measure PON-1 basal activity, in each plate well, 7 *μ*L of serum samples was added to a solution of 140 *μ*L of 0.1 mM Tris-HCl, 2 mM CaCl_2_, and 1.129 mM paraoxon (diethyl-4-nitrophenyl phosphate; Sigma Chemical, St. Louis, MO, USA). Six readings were taken at one-minute intervals in order to calculate PON-1 activity. The results were achieved by multiplying the mean absorbance variation by a calculated factor: factor = total reaction volume (TRV) (mL)/ε405-1805 mL^−1^ cm^−1^ × sample value (SV) (mL) × cuvette thickness (cm). Therefore, PON-1 activity = factor × Δabs/minute.

## 6. HDL Particle Size

The HDL particle size was measured by laser light scattering (LLS), using a ZetaPALS Zeta Potential analyzer with a 29 mW helium-neon laser at 658 nm (ZetaPALS; Brookhaven Instruments, Holtsville, NY) accordingly to the method described by Lima and Maranhão [10] at the Supercritical Nanotechnology Laboratory (LNS) from Polytechnic School, UFBA. 10 mL of blood was collected from each patient by a vacuum dispositive in a plastic tube (k_3_E k_3_-EDTA, VACUETTE®). Plasma was then obtained by centrifugation at 4°C for 15 min at 1.250 g. For the isolation of HDL and subsequent sizing by LLS, apoB-containing lipoproteins from each sample were precipitated by using a precipitating solution prepared with polyethylene glycol (PEG) 8000 (200 g/L) in 0.2 mol/L glycine buffer adjusted to pH 10 with NaOH. A 0.5 mL portion of each supernatant was added to 1.5 mL of NaCl (10 mmol/L), passed through a 0.22 *μ*m filter (Millipore Products Division), to exclude any undesirable particles, and then dispensed into a disposable cuvette. Light scattering (LS) was collected at a 90° angle by a photon-counting photomultiplier tube and then directed to a correlator, and consequently, BIC particle sizing's software derives particle sizes from the correlator function. Results of each sample were expressed as the mean, which is the harmonic intensity-averaged particle diameter. All experiments were performed at 25°C, and the results are expressed by the means of five runs of 2 min each.

## 7. Serum Free Cholesterol Determination

Free cholesterol from serum lipoproteins was determined by using an *in-house* total cholesterol reagent without cholesterol esterase activity (LABTEST Diagnostics S.A, Brazil). The free cholesterol absorbances were read in duplicate at 505 nm. Finally, to find free cholesterol concentration, the obtained absorbance was multiplied by the calculated factor. The HDL-esterified cholesterol was calculated as a difference between total and the measured free cholesterol multiplied by 1.67 [15].

## 8. Statistical Analysis

Statistical analysis was performed in two moments: first, the descriptive and then the inferential analysis. To test the type of data distribution, D'Agostino-Pearson's normality test was used. Grubb's test was performed to verify the presence of significative outliers before each comparison test. The comparison analysis was performed in columns, followed by the unpaired *t*-test, based on data distribution. For all statistical analyses, the tested parameters were considered significant when the critical “*p*” level was less than 0.05, for a 95% confidence interval.

## 9. Results

By comparing the participant's proportion between groups, the CP comparative group had similar sex participation of REAMI. The proportion of dyslipidemia and diabetes among REAMI patients was 40.6% and 5.85%, respectively. The proportion for the same conditions evaluated among CP participants was, respectively, 48% and 2.5%. The evaluated proportions of dyslipidemia and diabetes between groups were not significant. That clinical and laboratory information was obtained from the patient epidemiological questionnaire fulfillment at admission time in the Coronary Intensive Care Unit and at the ambulatory attendance at Faculty of Pharmacy Clinical and Toxicological Analysis Laboratory.

Table 1 shows the data comparison of lipid profile and lipid-related markers (i.e., total cholesterol, triglycerides, HDL-c, non-HDL-c, free cholesterol, and esterified cholesterol) from REAMI and CP groups determined in the study. Serum lipoproteins, total cholesterol, high-density lipoprotein cholesterol, and free cholesterol were significantly different between the evaluated groups.

**Figure 2:**
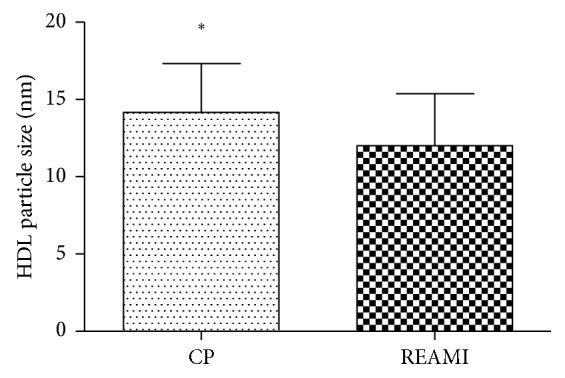
HDL particle size comparison between REAMI (*n*=43) and CP (*n*=40) groups. CP = nonischemic control patient, without complaints of an ischemic event. There was a significant difference between groups; REAMI was significantly different from CP (unpaired *t*-test; ^*∗*^*p* < 0.0001). A significant difference was accepted between groups when *p* < 0.05, for a 95% confidence interval.

Figure 2 shows the HDL particle size comparison between REAMI and CP groups. There was a significant difference between groups; the values are, respectively, 10.94 ± 0.23 nm and 13.59 ± 0.4 nm.

**Figure 3:**
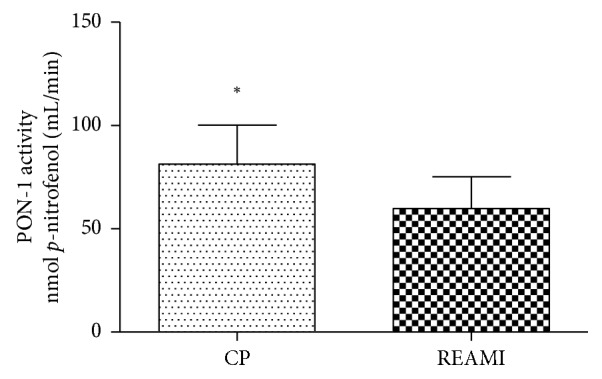
PON-1 activity comparison between REAMI (*n*=45) and CP (*n*=40) groups. CP = nonischemic control patients, a patient without complaints of an ischemic event. There was a significant difference among groups; REAMI was significantly different from CP (unpaired *t*-test; ^*∗*^*p*=0.0067). Significant difference among groups was accepted when *p* < 0.05, for a 95% confidence interval.

Similar to the comparison of HDL particle size between the CP and REAMI groups, the paraoxonase-1 activity also showed a significant difference, *p*=0.0067 (Figure 3). The values are, respectively, 90.5 ± 8.1 nmol/mL/min and 62.1 ± 6.3 nmol/mL/min.

The high sensitivity C-reactive protein (hs-CRP) concentrations between REAMI and CP groups behave significantly different (unpaired *t*-test; *p* < 0.0001). The median value of hs-CRP from CP was under the established cut-off reference value (2.08; 1.60–3.51). On the other hand, the REAMI patients, AMI compared group, had hs-CRP values above the reference limits (8.6; 3.4–15.0 mg/dL), median, and 25–75% confidence interval.

## 10. Discussion

The DATASUS-Brazil (2011) (http://www.datasus.gov.br/idb) showed that the ischemic heart disease mortality rate in males was 63.9% in all Brazilian states. Our study showed a 42% prevalence of male AMI patients and healthy participants. Regarding age, there was a higher prevalence of older patients from REAMI when compared to the nonischemic control patient (CP). Villela and collaborators [16] bring in their analysis a result very similar to ours in relation to the age and sex; according to them the proportional mortality related to CAD and cerebrovascular and hypertensive diseases was more prevalent in the age range between 60 and 69 in men and 70–79 years in women. Cardiovascular diseases are directly related to the presence of risk factors such as smoking, sedentary lifestyle, dyslipidemia, and diabetes mellitus [17]. Both groups participating in our study had similar dyslipidemia phenotype distribution, but differently, from REAMI, only one CP participant was diabetic. All diabetic participants had their glycemic levels controlled by oral hypoglycemic medication. According to Rubbo-Blanco et al. [18], patients with metabolic syndrome are at the highest risk of developing myocardial infarction, in addition to presenting higher total and cardiovascular mortality. Our findings show that serum lipoproteins free cholesterol was significantly high in CP participants. Although the compartmentalization of free and esterified cholesterol into lipoproteins is still something not well studied, Couto et al. [19] using LDE nanoparticles, a model of artificial lipoproteins, show that free cholesterol from lipoproteins are highly removed by the vascular wall of atherosclerotic patients compared to esterified cholesterol [19]. In addition to lipoprotein cholesterol compartmentalization, lipoproteins' structure diameter and particle size are associated with the quantities of free and esterified cholesterol into lipoproteins. In terms of HDL reverse cholesterol transport (RCT), the cholesterol compartmentalization is associated with lectin-cholesterol acyl-esterase (LCAT) and cholesteryl ester transfer protein (CETP) activities [20], transferring proteins associated with RCT and HDL size. According to our HDL particle size analysis, there was a significant difference between REAMI and CP groups. It was observed that the HDL particle diameter is higher in the CP group. Possibly, REAMI patients entered the emergency room with clinical suspicious of AMI in a stressful and inflammatory situation. Elbaz et al. [21] in a recent study report that HDL particles are continuously remodeled in terms of lipid and apolipoprotein composition since oxidative stress and inflammation can impair HDL functionality. By comparing HDL particle size from elderly patients with and without CAD, Azevedo et al. [22] found a significant diameter difference between groups, i.e., 9.7 ± 1.6 nm and 8.7 ± 0.7 nm, respectively (mean age 75 ± 6 years; *p*=0.0380), which corroborates our results. In addition, Parra et al. [23] who measured HDL diameters between 9.4 and 14 nm in a population with low CVD risk also found a positive association with CVD when considering HDL sizes between 7.3 and 8.2 nm.

Allied to the HDL particle size determination, our study also evaluated the paraoxonase-1 (PON-1) activity. The PON-1 activity showed a significant difference between REAMI and CP groups. It was observed that the PON-1 activity mean value in CP participants was higher. According to Correia and Perry [11], the PON-1 activity is lower in patients with carotid disease, coronary disease, and myocardial infarction, and therefore, it is related to heart disease risk. Bergmeier et al. [24] indicated that the higher the HDL density, the greater the PON-1 activity. Recent data have reinforced the view that HDL particle quality, structure, and function may better be represented by the relationship between HDL, as a surrogate marker, and the clinical event rather than the simple and isolated use of plasma HDL cholesterol information. Sentí et al. [25] also evaluated that the increased oxidative stress and the lower antioxidant effect may lead to metabolic syndrome with greater severity, causing metabolic oxidant-antioxidant imbalance.

The REAMI troponin and CK-MB mass results, as expected, were indicative of acute myocardial infarction (AMI). These patients entered the emergency room with characteristic chest pain and symptomatology. In addition to the troponin I and CK-MB mass results, as suggestive markers of myocardial injury, an electrocardiogram (ECG) was also performed as a complimentary exam to increase diagnostic sensitivity. Regarding the CRP determination, there were differences between the REAMI and CP groups. The CRP concentration reflected the inflammatory response, proving to be useful for patient's evaluation during ischemic processes. According to Teixeira et al. [26], CRP has an important role in the atherogenic mechanism because it is a nonspecific inflammatory acute phase protein. Sá et al. [27] performed CRP analysis in a cardiology emergency room and obtained a result equivalent to ours; according to them, patients with CAD previously documented by coronary angiography show higher median CRP concentrations compared to the control group. Although various studies show that CRP values are higher in the evaluation of patients with the previous history of cardiovascular disease, or in the presence of cardiovascular disease risk factors, there is no consensus among the scientific societies and governmental agencies, regarding the use of CRP for AMI evaluation, principally in patient admission as a cardiac emergency.

In summary, the inflammatory process acts by modulating negatively the lipid profile, and it acts in the reduction of lipoproteins synthesis by increasing the acute phase proteins production. According to Barbalho et al. [4], several authors showed the association between CRP concentration, metabolic syndrome, and the presence of atherosclerotic plaques. Higher values in plaque score and intimal average vessel thickness were found in individuals with metabolic syndrome. Volp et al. [28] reported that the ability of CRP to predict cardiovascular events has been described in the literature; i.e., those individuals with high CRP values, regardless of the severity of dyslipidemia, show a high risk of developing AMI.

In this way, we can see that there are components besides AMI that can influence the patient's baseline situation, such as stress, inflammatory process, capable of modifying HDL diameter (particle size), and PON-1 activity, which are measurements of HDL functionality. Therefore, it could be one of the possible answers why differences were found among the variables compared on REAMI and CP, patients with and without ACS symptomatology, and inflammatory process, respectively.

## 11. Conclusion

Despite an important current database on HDL cholesterol role, our study provides relevant complementary information about the HDL particle susceptibility to the inflammation following AMI. The HDL particles' quantitative and functional attributes, i.e. free and esterified cholesterol, particle size, and PON-1 antioxidant activity, should be measured and evaluated as markers of HDL functionality.

## Figures and Tables

**Table 1:** Comparison of REAMI and CP groups' lipid profile and other lipoprotein-derived serum biomarkers determined in the study.

	TC	TG	HDL-c	LDL-c	N-HDL-c	FC	EC
REAMI	177 ± 6.9	146 ± 14.2	41 ± 1.7	106 ± 5.9	135 ± 6.9	64 ± 3	189 ± 12
(*n*)	38	38	38	38	38	38	38
CP	203 ± 5.8	149 ± 10.7	61 ± 2.9	112 ± 5.2	141 ± 5.6	75 ± 3	208 ± 7
(*n*)	40	40	40	40	40	40	34
(*p*)	<0.005	ns	<0.0001	ns	ns	*p*=0.007	*p*=0.078

Results are expressed as mean and standard deviation. The unit mg/dL was adopted to lipid profile. TC = total cholesterol; TG = triglycerides; HDL-c = high-density lipoprotein cholesterol; LDL-c = low-density lipoprotein cholesterol; N-HDL-c = total cholesterol minus HDL cholesterol; FC = free cholesterol; EC = esterified cholesterol; ns = not significant. From the REAMI group (total participants: *n*=45), only 38 were evaluated, because seven patients missed lipid profile results. ^*∗*^Unpaired *t*-test, *p* < 0.05. Significant differences between groups when *p* < 0.05, for a 95% confidence interval.

## Data Availability

The data that support the findings of this study are available only with a reasonable request to the corresponding author (rdc@ufba.br). The data are not publicly available due to third-party restrictions, because the availability of these clinical and laboratory data was used under ethical license conditions applied for this study, which contained information that could compromise the privacy of research participants.

## References

[1] Palamalai V., Murakami M. M., Apple F. S. (2013). Diagnostic performance of four point of care cardiac troponin I assays to rule in and rule out acute myocardial infarction. *Clinical Biochemistry*.

[2] Huguenin F. M., Pinheiro R. S., Almeida R. M. V. R., Infantosi A. F. (2016). Characterization of the variation of health care taking into account the costs of hospital admissions for acute myocardial infarction in Brazilian Unified Health System. *Brazilian Journal of Epidemiology*.

[3] Thygesen K., Alpert J. S., White H. D. (2007). Universal definition of myocardial infarction. *Journal of the American College of Cardiology*.

[4] Barbalho S. M., Bechara M. D., Quesada K. (2015). Síndrome metabólica, aterosclerose e inflamação: tríade indissociável?. *Jornal Vascular Brasileiro*.

[5] Motta N. A. V., Fumian M. M., De Castro J. P. (2013). Inflammation and atherosclerosis: new biomarkers and therapeutic perspectives. *Brazilian Journal of Cardiology*.

[6] Lima E. S., Couto R. D. (2006). Estrutura, metabolismo e funções fisiológicas da lipoproteína de alta densidade. *Jornal Brasileiro de Patologia e Medicina Laboratorial*.

[7] Leança C. C., Passarelli M., Nakandakare E. R., Eder E. C. R. (2010). HDL: o yin-yang da doença cardiovascular. *Arquivos Brasileiros de Endocrinologia & Metabologia*.

[8] Quintão L., Chapman M. J., Kontush A. (2011). Biological activities of HDL subpopulations and their relevance to cardiovascular disease. *Trends in Molecular Medicine*.

[9] Santos A. P. C., Vieira M. S., Deus D. F. (2014). Atherogenic indices and HDL particle size as laboratory parameters to evaluate cardiovascular risk in the presence of dyslipidemia. *Journal of Biophysical Chemistry*.

[10] Lima E. S., Maranhão R. C. (2004). Rapid, simple Laser-Light-Scattering Method for HDL particle sizing in whole plasma. *Clinical Chemistry*.

[11] Correia J. D., Perry I. D. S. (2010). Dietary modulation of activity of paraoxonase: human studies review. *Revista HCPA*.

[12] Gugliucci A., Menini T. (2015). Paraoxonase 1 and HDL maturation. *Clinica Chimica Acta*.

[13] Charlton-Menys V., Liu Y., Durrington P. N. (2006). Semiautomated method for determination of serum paraoxonase activity using paraoxon as substrate. *Clinical Chemistry*.

[14] Gelisgen R., Genc H., Kayali R. (2011). Protein oxidation markers in women with and without gestational diabetes mellitus: a possible relation with paraoxonase activity. *Diabetes Research and Clinical Practice*.

[15] Neto O. G. L., Hueb W. A., Da Costa Sprandel M. O. (2014). Effects of glycemic control upon lipids and lipid transfers to HDL in patients with type 2 diabetes mellitus: novel findings in unesterified cholesterol status. *Experimental and Clinical Endocrinology & Diabetes*.

[16] Villela P. B., Klein C. H., Oliveira G. M. M. (2016). Trends in mortality from cerebrovascular and hypertensive diseases in Brazil between 1980 and 2012. *Arquivos Brasileiros de Cardiologia*.

[17] Teston E. F., Cecilio H. P. M., Santos A. L., Arruda G. O. D., Radovanovic C. A. T., Marcon S. S. (2016). Factors associated with cardiovascular diseases in adults. *Medicina (Ribeirao Preto. Online)*.

[18] Rubbo-Blanco M. L., Oliveira B. N., Filho A. G. S., Luiz R. R., Leão Lima R. D. S. (2015). Metabolic syndrome is the main predictor of myocardial ischemia in SPECT. *International Journal of Cardiovascular Sciences*.

[19] Couto R. D., Dallan L. A. O., Lisboa L. A. F., Mesquita C. H., Vinagre C. G. C., Maranhão R. C. (2007). Deposition of free cholesterol in the blood vessels of patients with coronary artery disease: a possible novel mechanism for atherogenesis. *Lipids*.

[20] Oliveira C. P., Maranão R. C., Bertato M. P., Wajchenberg B. L., Lerario A. C. (2012). Removal from the plasma of the free and esterified forms of cholesterol and transfer of lipids to HDL in type 2 diabetes mellitus patients. *Lipids in Health and Disease*.

[21] Elbaz M., Faccini J., Bongard V. (2016). High-density lipoprotein subclass profile and mortality in patients with coronary artery disease: results from the GENES study. *Archives of Cardiovascular Diseases*.

[22] Azevedo C. H. M., Wajngarten M., Lo Prete A. C. (2011). Simultaneous transfer of cholesterol, triglycerides, and phospholipids to high-density lipoprotein in aging subjects with or without coronary artery disease. *Clinical Science*.

[23] Parra E. S., Panzoldo N. B., Zago V. H. (2014). HDL size is more accurate than HDL cholesterol to predict carotid subclinical atherosclerosis in individuals classified as low cardiovascular risk. *PLoS One*.

[24] Bergmeier C., Siekmeier R., Gross W. (2004). Distribution spectrum of paraoxonase activity in HDL fractions. *Clinical Chemistry*.

[25] Sentí M., Tomás M., Fitó M. (2003). Antioxidant Paraoxonase 1 activity in the metabolic syndrome. *Journal of Clinical Endocrinology & Metabolism*.

[26] Teixeira D. A., De Souza C. F. P., Pereira G. L. H. (2009). C-reactive protein: association between inflammation and complications after acute myocardial infarction in the elderly. *Revista Brasileira de Clínica Médica*.

[27] Sá M. P. B. O., Gomes R. A. F., Santos T. O. C. (2009). C-reactive protein of high sensitivity in patients with acute myocardial infarction in the cardiological emergency. *Revista Brasileira de Clínica Médica*.

[28] Volp A. C. P., Alfenas R. d. C. G., Costa N. M. B., Minim V. P. R., Stringueta P. C., Bressan J. (2008). Capacidade dos biomarcadores inflamatórios em predizer a síndrome metabólica: inflammation biomarkers capacity in predicting the metabolic syndrome. *Arquivos Brasileiros de Endocrinologia & Metabologia*.

[29] Faludi A. A., Izar M. C. O., Saraiva J. F. K. (2017). Atualização da diretriz brasileira de dislipidemias e prevenção da aterosclerose–2017. *Arquivos Brasileiros de Cardiologia*.

